# Face Distortion Aftereffects Evoked by Featureless First-Order Stimulus Configurations

**DOI:** 10.3389/fpsyg.2012.00566

**Published:** 2012-12-17

**Authors:** Pál Vakli, Kornél Németh, Márta Zimmer, Stefan R. Schweinberger, Gyula Kovács

**Affiliations:** ^1^Department of Cognitive Science, Budapest University of Technology and EconomicsBudapest, Hungary; ^2^Department of General Psychology and Cognitive Neuroscience, Friedrich Schiller University of JenaJena, Germany; ^3^DFG Research Unit Person Perception, Friedrich Schiller University of JenaJena, Germany; ^4^Institute of Psychology, Friedrich Schiller University of JenaJena, Germany; ^5^Institute of Psychology, University of RegensburgRegensburg, Germany

**Keywords:** face distortion aftereffect, first-order relations, second-order relations, configural processing, contrast polarity

## Abstract

After prolonged exposure to a distorted face with expanded or contracted inner features, a subsequently presented normal face appears distorted toward the opposite direction. This phenomenon, termed as face distortion aftereffect (FDAE), is thought to occur as a result of changes in the mechanisms involved in higher order visual processing. However, the extent to which FDAE is mediated by face-specific configural processing is less known. In the present study, we investigated whether similar aftereffects can be induced by stimuli lacking all the typical characteristics of a human face except for its first-order configural properties. We found a significant FDAE after adaptation to a stimulus consisting of three white dots arranged in a triangular fashion and placed in a gray oval. FDAEs occurred also when the adapting and test stimuli differed in size or when the contrast polarity of the adaptor image was changed. However, the inversion of the adapting image as well as the reduction of its contrast abolished the aftereffect entirely. Taken together, our results suggest that higher-level visual areas, which are involved in the processing of facial configurations, mediate the FDAE. Further, while adaptation seems to be largely invariant to contrast polarity, it appears sensitive to orientation and to lower level manipulations that affect the saliency of the inner features.

## Introduction

In the course of the last decade, several studies have demonstrated that the way we perceive faces is systematically biased by the characteristics of a previously presented face, a phenomenon commonly referred to as the face adaptation aftereffect (FAE). A prime example of such face – related aftereffects is the so-called face distortion aftereffect (FDAE): following adaptation to a distorted face, a subsequently presented normal face appears distorted in the opposite way (O’Leary and McMahon, 1991; Webster and MacLin, 1999; MacLin and Webster, 2001). For example, an undistorted face seems expanded after viewing a face with features compressed toward the midline. Besides distortion, FAEs have been observed for a number of natural facial properties including identity (Leopold et al., 2001), gender (Webster et al., 2004), age (Schweinberger et al., 2010), ethnicity (Webster et al., 2004) as well as more dynamic facial features such as emotional expression (Webster et al., 2004; Fox and Barton, 2007), eye-gaze direction (Jenkins et al., 2006; Seyama and Nagayama, 2006), and lip angle (Jones et al., 2010).

Such perceptual aftereffects enable researchers to link changes in perception to changes in the underlying neural mechanisms and thus provide information about the representation of complex visual patterns in the brain. One fundamental question about FAEs is the extent to which they reflect the recalibration of neural populations engaged in high-level visual processing. Since the rationale behind adaptation is that the same or overlapping neural populations process the adaptor and test stimuli, the tolerance of FAEs toward physical differences between the adaptor and test images provides important clues about the neural locus of the aftereffects. For example, it has been shown that although the magnitude of the FDAE is the greatest when the images are of the same size, the aftereffect survives a two-octave difference in size between adaptor and test faces (Zhao and Chubb, 2001). Aftereffects for facial identity are also tolerant to differences in image size (Leopold et al., 2001; Anderson and Wilson, 2005), and the size-invariance of the face identity aftereffect can be observed in younger age as well (Pimperton et al., 2009). These results are in line with data from monkey single-cell recordings (Perrett et al., 1982; Rolls and Baylis, 1986) and functional brain imaging studies in humans (Andrews and Ewbank, 2004) demonstrating a largely size-invariant neural representation of faces in the ventral regions of the temporal lobe.

FAEs have also been shown to transfer across different retinal positions (Leopold et al., 2001; Fang and He, 2005), albeit they are not entirely position-invariant (Kovács et al., 2005), and the magnitude of the aftereffect decreases with increasing distance between the adaptor and test stimuli (Afraz and Cavanagh, 2008). To date, the results regarding the position-sensitivity of FAEs have been controversial, with studies emphasizing the contribution either of spatiotopic (Melcher, 2005; van Boxtel et al., 2008) or of retinotopic coding (Afraz and Cavanagh, 2009). These inconsistencies may result from the different adaptation protocols (identity-specific versus gender-specific) employed in the above-mentioned studies, which are thought to tap different cortical processing sites (see Zimmer and Kovács, 2011b for a review). In addition, the duration of the adaptation period is a critical factor that determines the position-sensitivity of the aftereffect, since varying the time course of adaptation allows one to selectively adapt position-sensitive and position-invariant neural populations along the ventral visual pathway (Kovács et al., 2007, 2008).

Besides position, FAEs also tolerate remarkable differences in picture plane orientation and viewpoint between the adaptor and test faces. For example, Watson and Clifford (2003) have shown that the FDAE rotates with the test face in the picture plane, suggesting that the distortion is coded in an object-based reference frame. In relation to three-dimensional orientation, it has been shown that FAEs induced in one viewpoint transfer to other viewpoints, although this transfer is limited in a sense that the aftereffect decreases as the angular difference between the adaptor and test views increases (Benton et al., 2006, 2007; Jeffery et al., 2006). This finding can be explained in terms of viewpoint-specific coding, subserved by face-selective areas in the ventral visual cortex, which show viewpoint-sensitive fMRI adaptation as well (Fang et al., 2007).

Taken together, these results suggest that FAEs reflect the adaptation of neural populations at higher-levels of the visual processing stream that tolerate substantial changes in several low-level attributes of the stimulus, such as retinal size, position, and viewpoint. This notion is further supported by studies showing that aftereffects of identity are not affected by differences in facial expression between the adaptor and test stimuli (Fox et al., 2008), or the distortion of the adaptor face by vertical stretching (Hole, 2011), which implies that the adaptation affects a rather abstract representation of facial identity (Hole, 2011). On the other hand, the extent to which these aftereffects are mediated by processing sites that are sensitive to the configural properties of faces is a matter of further inquiry. The term “configural processing” in the face perception literature refers to the encoding of the exact relations among the constituent elements of the face (Maurer et al., 2002). This process involves the detection of the basic configuration that all faces share, that is, the relative position of the eyes, nose, and mouth (first-order relations) as well as the encoding of the precise metric distances among the features (second-order relations – Diamond and Carey, 1986; Maurer et al., 2002). A related phenomenon that is often used interchangeably with configural processing is “holistic processing,” which refers to the integration of the features as well as their spatial relations in a single unified representation that makes the processing of individual features rather difficult (Young et al., 1987; Tanaka and Farah, 1993, for a recent review, see Tanaka and Gordon, 2011). The contribution of configural/holistic processing to face perception can be demonstrated for example by the face inversion effect – the disproportionate detriment in our ability to recognize faces as opposed to objects when they are presented upside-down (Yin, 1969). Since inversion affects face recognition more than the recognition of objects, it is thought to tamper with perceptual mechanisms that are unique to face processing. Indeed, impoverished recognition of inverted faces is attributed to the diminished performance in detecting fine-scale differences in the metric distances among facial features (e.g., Sergent, 1984; Searcy and Bartlett, 1996; Freire et al., 2000), which is thought to be in connection with the inability to integrate distant elements of the face into a unified percept (Rossion, 2008, 2009, however, there is an alternative view according to which inversion disrupts the coding of individual features as well, as long as featural information is defined in terms of variations in shape, see McKone and Yovel, 2009 for a review).

Therefore, the face inversion effect is a useful behavioral marker of configural/holistic processing, which operates normally when the visual system is presented with an upright face, but breaks down when the face is turned upside-done. It follows from this that if FAEs reflect the adaptation of neural populations engaged in such mechanisms, they should also be sensitive to inversion. However, in many cases, the aftereffects observed with both the adaptor and test faces turned upside-down are of the same magnitude as those reported when the adaptor and test faces are upright. (Webster and MacLin, 1999; Leopold et al., 2001; Zhao and Chubb, 2001; Watson and Clifford, 2003, 2006; Guo et al., 2009; but see Rhodes et al., 2009a). On the other hand, aftereffects do not transfer fully between faces in opposite orientations (Webster and MacLin, 1999), and this is especially true when the adaptor face is inverted and the test face remains upright (Watson and Clifford, 2003, 2006; Guo et al., 2009). One possible explanation for this asymmetry is that aftereffects following adaptation to upright and inverted faces arise at different stages of the visual system – adaptation to upright faces affect both face-specific configural/holistic representations and non-specific part-based representations, whereas adaptation to inverted faces affects only the later (Watson and Clifford, 2003, 2006). The assumption that adaptation to upright and inverted faces tap into different representations finds support from orientation-contingent aftereffects, that is, opposite aftereffects are induced for upright and inverted faces at the same time (Rhodes et al., 2004). A related notion is that upright face aftereffects reflect partly, while inverted aftereffects reflect entirely the recalibration of high-level generic shape-coding mechanisms (Susilo et al., 2010). Susilo et al. (2010) found that aftereffects for eye-height show a partial transfer between T-shapes and real faces. For example, adaptation to upright T-shapes resulted in an aftereffect in eye-height judgments of upright real faces, but this aftereffect was smaller than the one obtained by real face adaptors. In contrast, there was a complete transfer between the two stimulus classes when they were presented upside-down. These findings can be taken as evidence that a shape-generic component can partly account for upright face aftereffects. Another factor that appears to modulate the transfer of aftereffects between adaptor and test faces of opposite orientation is familiarity. Hills and Lewis (2012) found that identity aftereffects for famous faces showed greater transfer from inverted adaptors to upright images than vice versa. This pattern is the exact opposite of the ones observed in FDAEs and face gender aftereffects with unfamiliar faces (Watson and Clifford, 2003, 2006). Since the FDAE and the identity aftereffects are usually assumed to reflect the operation of the same mechanisms (Hurlbert, 2001; Webster and MacLeod, 2011), the above discrepancy is rather attributable to the effect of familiarity than to the different types of aftereffects examined in these studies (Hills and Lewis, 2012).

Turning to the role of basic facial configuration (first-order spatial relations) in FAEs, it has been shown that an adaptor with a preserved whole-face configuration is crucial for identity aftereffects (Pichler et al., 2012), but not for aftereffects of facial affect (Butler et al., 2008). However, the latter can also be induced with adaptors consisting of non-facial elements, provided that they are arranged in a face-like fashion (Butler et al., 2008). Thus, it seems that in both cases, the locus of adaptation is sensitive to the basic geometrical structure of the face. In addition, the identity aftereffect showed a significant decrease in magnitude when the adaptor and test faces differed in the metric distances between their features (Pichler et al., 2012).

**Figure 1:**
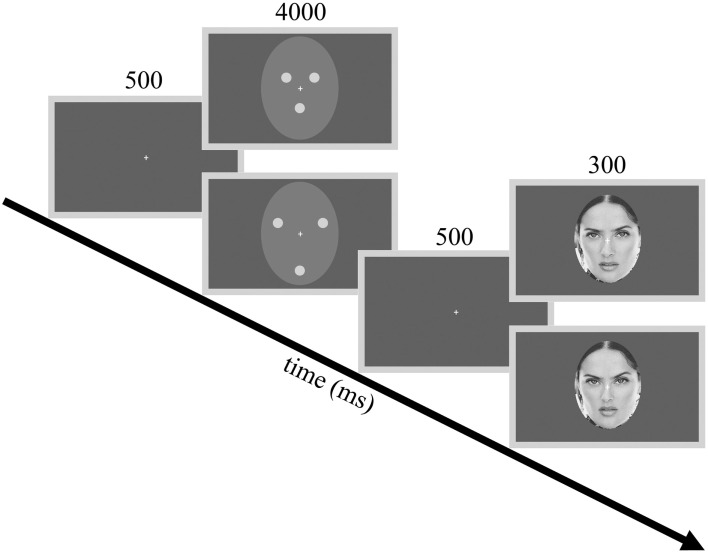
**Procedures and example stimuli**. The flowchart illustrates the adaptor stimuli used in Experiment 1 and one of the three test faces used during the experiments as an example. Adaptor stimuli from top to down: contracted (CONT) and expanded (EXP) white dots. Test stimuli from top to down: −10% (expanded) and +10% (contracted) distorted faces.

The above results emphasize the role of first- and second-order spatial relations in upright face aftereffects of facial identity and emotion. Previous studies examining the effect of inversion on FAEs (see above) suggested that these facial properties might also be important for aftereffects of gender and distortion. In case of distortion, a recent study has shown that the FDAE is contingent on emotional expression and gender, which might indicate that the underlying processing sites are sensitive to configural changes that differentiate between faces varying along these dimensions (Tillman and Webster, 2012). However, as the authors note, these results can be explained by the adaptation of processing sites engaged in more generic visual processing, and do not necessarily involve face-specific response changes. Therefore, unraveling the precise nature of the representations underlying these aftereffects requires further investigation. In the present study, our aim was to investigate the role of basic facial configuration in the FDAE. We used schematic face-like images as adaptor stimuli that preserve the basic configural properties of a face (the first-order spatial relations of the major parts) but only consist of simple geometric shapes and therefore lack the typical features that describe a real human face (Figure 1). Previous studies have shown that newborns orient preferentially to such stimuli (Johnson et al., 1991) and that in adults, schematic faces activate a face-selective cortical area, the fusiform face area (FFA – Kanwisher et al., 1997) more strongly than non-face objects, albeit less strongly than real faces do (Tong et al., 2000; Liu et al., 2009). Photographs of real faces of famous celebrities with different degrees of distortion (expansion and contraction) served as target stimuli in our experiments. We argued that if the processing sites underlying the FDAE are sensitive to the basic configural properties of a face, then they should be activated by the schematic face-like adaptors. If this were so, then prolonged exposure to these adaptors with variations in the metric distances between their components (expanded or contracted face-like stimuli) would bias the perception of the subsequently presented real faces, resulting in a systematic aftereffect. In other words, we tested whether the FDAE can be induced with simple geometric shapes arranged in a face-like pattern (Experiment 1). We also assessed whether such an aftereffect reflects the adaptation of a high-level processing sites by manipulating several low-level features of the adaptor stimuli, such as size (Experiment 2), orientation (Experiment 3), contrast polarity (Experiment 4), and the effect of replacing the constituent elements with visual noise (Experiment 5).

## Experiment 1

### Materials and methods

#### Participants

Thirteen naive, healthy volunteers (six females) participated in the experiment (mean age: 26 ± 3 years). All the participants had normal or corrected-to-normal vision and gave written informed consent. We conform to the protocols approved by the Ethical Committee of the University of Regensburg.

#### Stimuli

We used the full-front gray-scale face images of three famous persons (Angelina Jolie, Nicole Kidman, and Salma Hayek) as test faces. These faces were compressed and expanded using the Adobe Photoshop 6.0 “Pinch” option. We applied four different expansion (−20%, −15%, −10%, −5%) and four different contraction (5%, 10%, 15%, 20%) levels to the face images. These distortions affected the shape of the internal features of the face as well as their exact spatial relations while the outer contour of the face and the overall shape of the head remained the same (Zimmer and Kovács, 2011a). The three undistorted celebrity faces and their four expanded and four contracted versions (corresponding to the distortion levels described above) were used as test faces. Thus, there were a total of 27 face images that served as test stimuli in the present experiment and in all the other experiments reported in this paper.

Two different adaptation conditions were presented in separate blocks. In both conditions, the adaptor image consisted of three white dots (luminance: 64 cd/m^2^), arranged in a triangular fashion. The dots were placed according to the location of the eyes and mouth and were embedded in a light gray elliptic surround (luminance: 13 cd/m^2^, Michelson contrast = 0.66). The elliptic surround subtended a visual angle of 9°× 11 under a viewing distance of 70 cm. In the contracted adaptor condition (CONT), the distance between the individual dots was 2.1°. In the expanded adaptor condition (EXP), the space between the dots was increased to 3.9° (Figure 1). Stimuli were presented in the center of the screen on a uniform gray background using a 17″ monitor (1024 × 768 pixel resolution, 75 Hz vertical refresh rate). Participants were tested individually in a dimly lit room. All software was written in MATLAB 6.5 (MathWorks, Inc.) using PsychToolbox 2.45 for Windows.

#### Procedure

Before the beginning of the test phase, participants were familiarized with each celebrity whose images were used as target stimuli in the test phase. During this “familiarization phase,” participants were presented with the veridical, 20% contracted and 20% expanded images of each of the three celebrities and they were asked to note the differences between the original and the distorted images, as well as to recognize these persons and recall their names.

The testing phase followed a course that was similar to that of Zimmer and Kovács (2011a). In the beginning of each trial, a blank screen appeared for 500 ms followed by the adaptor image, which was presented for 4000 ms. Following the adaptor image there was a 500 ms gap, after which the test stimulus was presented for 300 ms. Participants were instructed to fixate on a white crosshair presented centrally on the screen and to press a button whenever they perceived the test face expanded or another button if the test face appeared contracted compared to the veridical, undistorted face of the given celebrity. Contracted and expanded adaptor conditions of all the three celebrities were given in two separate blocks, with a short break between the two. The order of the blocks was randomized across participants. Each block consisted of 135 trials – 9 (number of distortion levels) x 3 (number of celebrities) × 5 (number of repetitions of a given test stimulus) – in a random order. Experimental sessions lasted approximately 30 min.

#### Data analysis

Psychophysical data were modeled by the Weibull psychometric function, using the Psignifit toolbox (Version 2.5.6.) for MATLAB (Wichmann and Hill, 2001). In order to determine whether adaptation to contracted or expanded dot patterns results in a bias in face distortion discrimination of the subsequently presented target stimuli, we conducted a two-way repeated measures analysis of variance (ANOVA) with type of adaptor (2) and distortion level (9) as within-subject factors.

### Results and comment

Participants’ contraction ratings varied with different levels of distortion, indicating that they perceived the negative and positive distortions of the target faces [main effect of distortion level: *F*(8, 96) = 34.21, *p* < 0.0001, $\eta_{p}^{2}=0.74$]. Another observable tendency is that on average, participants perceived the test faces to be more expanded than contracted. Specifically, at 0% distortion (veridical face), the percentage of “contracted” ratings is slightly less than 50%, even in the expanded adaptor condition. One factor that might have contributed to this effect is the sensitivity to different directions of distortion, namely that people are more sensitive to inwards than outwards shifts of the eyes (Haig, 1984). This might have counteracted the aftereffect to the extent that the veridical face was reported somewhat more often as “expanded” than “contracted.”

**Figure 2:**
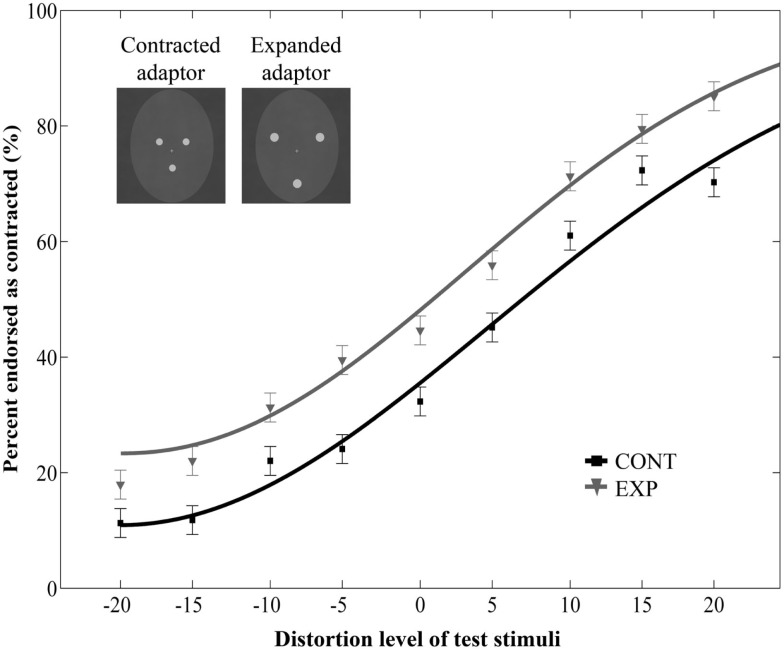
**Mean ratio of stimuli endorsed as contracted as a function of distortion level (% distorted)**. Negative and positive distortion levels correspond to expanded and contracted target faces respectively. Results obtained by using contracted (CONT) and expanded (EXP) white dots as adaptor stimuli. The inset illustrates the adaptor stimuli. Data are modeled by a Weibull psychometric function.

More importantly, adaptation to the dot patterns biased the perception of the target faces, causing a significant aftereffect: test faces were judged more contracted following adaption to an expanded, than to a contracted dot pattern [main effect of adaptor type: *F*(1, 12) = 38.92, *p* < 0.0001, $\eta_{p}^{2}=0.76,$ no interaction between adaptor type and distortion level: *F*(8, 96) = 0.43, *p* = 0.9, $\eta_{p}^{2}=0.03,$ Figure 2]. This indicates that perceptual aftereffects for faces can be induced by using relatively simple adaptor stimuli, such as three dots arranged in a face-like fashion. Moreover, the pattern of results suggests that these aftereffects are similar in nature to those reported in previous studies demonstrating that prolonged viewing of a distorted face biases the perception of a subsequently presented face in a way that is opposite to the distortion of the adaptor image (Webster and MacLin, 1999; Zhao and Chubb, 2001; Watson and Clifford, 2003; Yamashita et al., 2005; Zimmer and Kovács, 2011a). The obvious differences in terms of physical characteristics between the adaptor and the test stimuli used in the present experiment suggest that the neural mechanisms of this aftereffect are not engaged in image-based, but rather in higher-level visual processing. To investigate the contribution of such high-level adaptation, we developed another experiment in which the adaptor and test stimuli differed in size. Since there is considerable evidence for a size-invariant neural representation of faces in both monkeys (Perrett et al., 1982; Rolls and Baylis, 1986) as well as in humans (Andrews and Ewbank, 2004), we hypothesized that if the aftereffect is indeed mediated by high-level visual areas, then it would occur despite a remarkable difference in size between the adaptor and test images (Leopold et al., 2001; Zhao and Chubb, 2001; Anderson and Wilson, 2005; Pimperton et al., 2009).

## Experiment 2 – Size

### Materials and methods

A new group of 11 naive, healthy participants (five females, mean age: 26 ± 4 years) with normal or corrected-to-normal vision participated in the experiment and gave written informed consent. In this experiment, the adaptor and test images were identical to those of Experiment 1 except that the adaptor stimuli were 30% larger than the test faces. To compare the results of the present experiment to those of Experiment 1, we analyzed the data from both experiments together in a three-way mixed-design ANOVA with size (2; same/different) as a between-subject factor and adaptor type (2) and distortion level (9) as within-subject factors.

### Results and comment

Prolonged exposure to the dot pattern resulted in an aftereffect: adaptation to a dot pattern distorted in one way caused the subsequent test faces to appear distorted in the opposite way [main effect of adaptor type: *F*(1, 22) = 29.18, *p* < 0.0001, $\eta_{p}^{2}=0.57,$ main effect of distortion level: *F*(8, 176) = 72.83, *p* < 0.0001, $\eta_{p}^{2}=0.77,$ no interaction between adaptor type and morph level: *F*(8, 176) = 1.2, *p* = 0.3, $\eta_{p}^{2}=0.05$]. Crucially, the main effect of size was not significant [*F*(1, 22) = 0.6, *p* = 0.45, $\eta_{p}^{2}=0.03$], and there was no interaction between size and adaptor type [*F*(1, 22) = 1.62, *p* = 0.22, $\eta_{p}^{2}=0.07$]. The three-way interaction was also not significant [*F*(8, 176) = 0.7, *p* = 0.69, $\eta_{p}^{2}=0.03$]. These results suggest that aftereffects occur also when the schematic face-like adaptors and the test faces differ in size.

**Figure 3:**
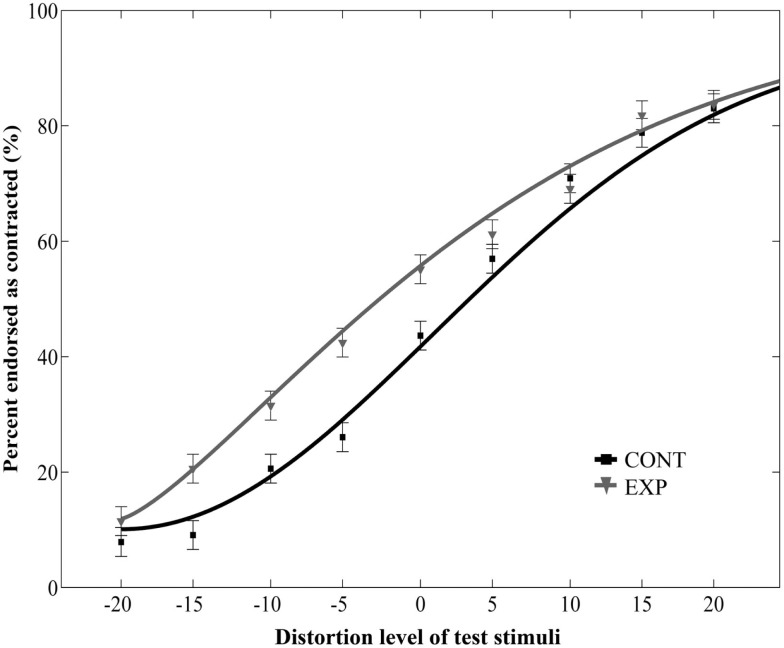
**Mean ratio of stimuli endorsed as contracted as a function of distortion level (% distorted) when the relative size of adaptor and target was varied**. Negative and positive distortion levels correspond to expanded and contracted target faces respectively. Results obtained by using versions of contracted (CONT) and expanded (EXP) white dot adaptors that differed in size from the target stimuli.

Additionally, we ran a separate two-way repeated measures ANOVA on the data of the Experiment 2 with adaptor type (2) and distortion level (9) as within-subject factors. This analysis yielded a significant main effect of adaptor type [*F*(1, 10) = 5.43, *p* = 0.04, $\eta_{p}^{2}=0.35$] and distortion level [*F*(8, 80) = 39.36, *p* < 0.0001, $\eta_{p}^{2}=0.8,$ no interaction between adaptor type and distortion level: *F*(8, 80) = 1.34, *p* = 0.24, $\eta_{p}^{2}=0.12,$ Figure 3]. Taken together, these results show that the aftereffect tolerates remarkable size differences between the adaptor and test images.

The fact that the aftereffect is, to a great extent, size-invariant suggests that it is mediated by higher processing levels of the visual system. However, the degree to which the aftereffect is due to the adaptation of a neural population involved in face-specific configural processing requires further investigation. To this end, we conducted an additional experiment in which the dot pattern was inverted while the orientation of the test images remained upright. It is well known that turning a face upside-down deteriorates its recognition greatly (Yin, 1969). This so-called “face inversion effect” is believed to arise due to the disruption of coding the spatial relations between face elements and thus regarded as the hallmark of configural processing (Maurer et al., 2002; Rossion and Gauthier, 2002; see Introduction). Thus, inverting the dot pattern presumably renders it more difficult to encode its face-like configural properties. Therefore we hypothesized that if face-sensitive processing sites account for the aftereffects observed in Experiment 1 and 2, then the inversion of the adaptor image should reduce or even eliminate the aftereffect.

## Experiment 3 – Orientation

### Materials and methods

A new group of 10 participants (nine females, mean age: 22 ± 3 years) with normal or corrected-to-normal vision was recruited for the experiment and gave written informed consent. Task instructions, adaptor and test stimuli were the same as in Experiment 1, except that the adaptor images were turned upside-down. To compare the results of the present experiment to those of Experiment 1, we analyzed the data from both experiments together in a three-way mixed-design ANOVA with orientation (2; upright/inverted) as a between-subject factor and adaptor type (2) and distortion level (9) as within-subject factors.

### Results and comment

The main effect of adaptor type was significant [*F*(1, 21) = 20.22, *p* = 0.0002, $\eta_{p}^{2}=0.49$] but it was qualified by a significant interaction between adaptor type and orientation [*F*(1, 21) = 8.75, *p* = 0.007, $\eta_{p}^{2}=0.29$]. *Post hoc* tests (Fisher’s Least Significant Difference test) revealed that contracted ratings significantly differed between CONT and EXP conditions in case of upright adaptors (*p* < 0.0001), whereas there was no such difference in case of inverted adaptors (*p* = 0.32). The main effect of orientation [*F*(1, 21) = 1.45, *p* = 0.24, $\eta_{p}^{2}=0.06$] and the three-way interaction [*F*(8, 168) = 1.45, *p* = 0.93, $\eta_{p}^{2}=0.02$] were not significant. Thus, while aftereffects were observed with upright adaptors, the inversion of the adaptor stimuli eliminated the aftereffect. We also observed a main effect of distortion level [*F*(8, 168) = 113.05, *p* < 0.0001, $\eta_{p}^{2}=0.84$] and an interaction between distortion level and orientation [*F*(8, 168) = 4.29, *p* = 0.0001, $\eta_{p}^{2}=0.17$], but no interaction between distortion level and adaptor type [*F*(8, 168) = 0.82, *p* = 0.59, $\eta_{p}^{2}=0.04$].

**Figure 4:**
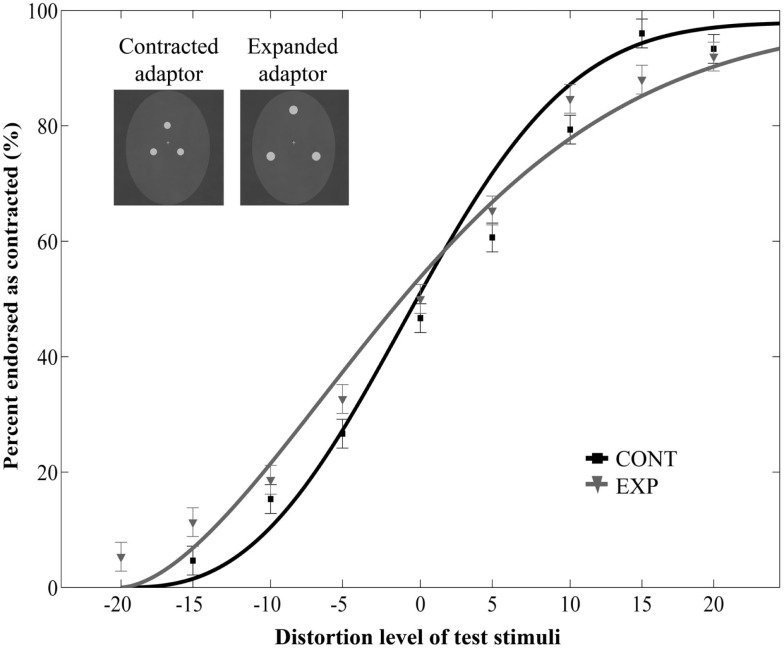
**Mean ratio of stimuli endorsed as contracted as a function of distortion level (% distorted) when the adaptor images were upside-down**. Negative and positive distortion levels correspond to expanded and contracted target faces respectively. Results obtained by using the inverted versions of contracted (CONT) and expanded (EXP) white dots as adaptors.

Additionally, we ran a separate two-way repeated measures ANOVA on the data of Experiment 3 with adaptor type (2) and distortion level (9) as within-subject factors. This analysis yielded to no significant effect of adaptor type [*F*(1, 9) = 0.85, *p* = 0.38, $\eta_{p}^{2}=0.09$ with a significant main effect of distortion: *F*(8, 72) = 110, *p* < 0.0001, $\eta_{p}^{2}=0.92$ and to no interaction of adaptor type and distortion: *F*(8, 72) = 0.71, *p* = 0.68, $\eta_{p}^{2}=0.07,$ Figure 4]. Thus, prolonged viewing of inverted dot patterns did not bias the perception of test faces.

This result implies that the exact configuration of the dot pattern applied in Experiment 1 – three dots arranged in a triangular fashion – is crucial to evoke the aftereffect. Since this arrangement mimics the first-order configural properties of a face, the lack of aftereffect when the dot pattern is inverted suggests the involvement of face-sensitive configural processing sites. Whether these processing sites represent faces based solely on configural information, or whether they are sensitive to low-level cues remains an open issue. Hence, we conducted an additional experiment to test the role of low-level features in which the contrast polarity of the adaptor image was varied by presenting either white dots on a black background or black dots on a white background. We reasoned that if the adapting sites are sensitive solely to configural properties, then aftereffects should be obtained irrespective of the actual contrast polarity of the adaptor images.

## Experiment 4 – Contrast Reversal

### Materials and methods

Ten participants (six females, mean age: 29 ± 8 years) with normal or corrected-to-normal vision participated in the experiment and gave written informed consent. Task instructions, test stimuli, and overall procedures were identical to those of Experiment 1. The adaptors either consisted of white dots on a black oval, or black dots on a white oval. In both cases, the contrast between the dots and the oval was the same (Michelson contrast = 0.95). Both types of adaptors appeared in two forms: expanded and contracted to the same extent as in Experiment 1. Thus, there were a total of four conditions (expanded and contracted white dot adaptors; expanded and contracted black dot adaptors). Each participant was tested with all four adaptors with the order of the conditions randomized across participants. A three-way repeated measures ANOVA was employed to determine the effects of adaptation on the distortion discrimination of the test faces, with contrast polarity (2), adaptor type (2) and distortion level (9) as within-subject factors.

### Results and comment

**Figure 5:**
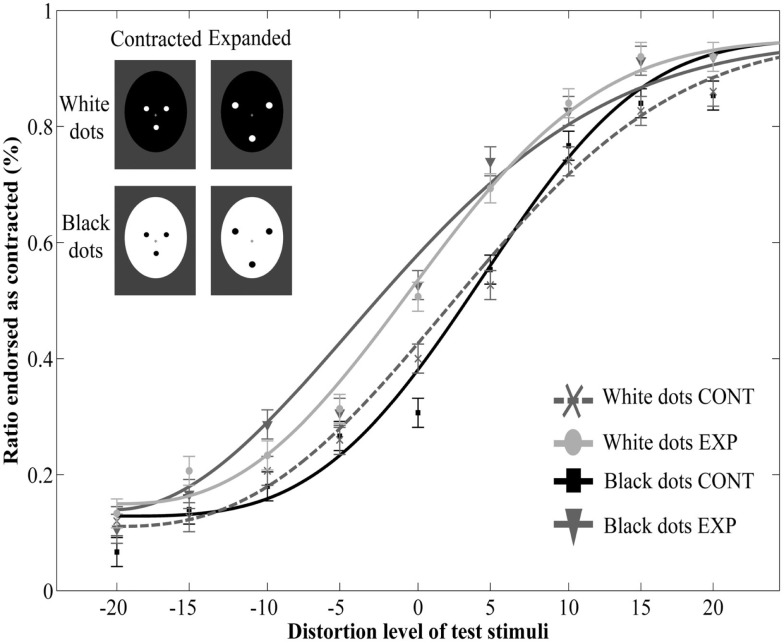
**Mean ratio of stimuli endorsed as contracted as a function of distortion level (% distorted) with adaptors of opposite contrast polarity**. Negative and positive distortion levels correspond to expanded and contracted target faces respectively. Results obtained by using white dots on a black oval (White dots CONT and White dots EXP) and black dots on a white oval (Black dots CONT and Black dots EXP).

The main effect of adaptor type was significant [*F*(1, 9) = 6,76, *p* = 0.029, $\eta_{p}^{2}=0.43$], showing that adaptation to the dot patterns resulted in a perceptual aftereffect. However, neither the main effect of polarity [*F*(1, 9) = 0.06, *p* = 0.82, $\eta_{p}^{2}=0.006$], nor the interaction between polarity and adaptor type [*F*(1, 9) = 0.33, *p* = 0.58, $\eta_{p}^{2}=0.0$] was significant. The three-way interaction between polarity, adaptor type and distortion level was also not significant [*F*(8, 72) = 0.75, *p* = 0.65, $\eta_{p}^{2}=0.08$]. Finally, there was a significant main effect of distortion level [*F*(8, 72) = 42.86, *p* < 0.0001, $\eta_{p}^{2}=0.83$], while every other effect was non-significant (p values above 0.16, Figure 5).

These results show that prolonged viewing of contracted and expanded adaptors results in a perceptual aftereffect similarly to the findings of Experiment 1 and 2. The results also show that when the internal elements of the adaptor image are matched in contrast (hence in perceptual saliency), this effect does not depend on the contrast polarity of the adaptor image. These findings indicate that the underlying processing sites represent the structural properties of the image largely independently of contrast polarity.

However, it is possible that the adaptation sites are sensitive to other low-level manipulations that affect the saliency of the internal features of the adaptor. Hence we investigated to role of low-level image properties in a further experiment in which we used the upright contracted and expanded adaptor images with their constituent dots replaced by equiluminant visual noise patterns, reducing the contrast between the dots and their background strongly. We reasoned that if the locus of adaptation is sensitive to the contrast of the constituent elements, then replacing these elements with visual noise should also reduce or eliminate the aftereffect.

## Experiment 5 – Low-Contrast

### Materials and methods

Eleven participants (10 females, mean age: 27 ± 4 years) with normal and corrected-to-normal vision participated in the experiment and gave written informed consent. Task instructions, test stimuli and overall procedure were the same as in Experiment 1. Adaptor images had the same configuration as those in Experiment 1 but their constituent elements were replaced by visual noise. First, Fourier phase-randomization was applied to the original versions of the three celebrity faces. Second, the resulting images were equated in luminance (13 cd/m^2^) and were resized to match the size of the dots of the adaptor stimulus. Finally, the three noise patterns were placed on a gray oval (luminance: 8 cd/m^2^) at the locations corresponding to the eyes and mouth of a face, as in the previous experiments. The contrast between the dots and the oval background was reduced strongly (Michelson contrast = 0.24) when compared to the previous experiments. To compare the results of the present experiment to those of Experiment 1, we analyzed the data from both experiments together in a three-way mixed-design ANOVA with dot quality (2; white dots/noise) as a between-subject factor and adaptor type (2) and distortion level (9) as within-subject factors.

### Results and comment

The main effect of adaptor type was significant [*F*(1, 22) = 16.43, *p* = 0.0005, $\eta_{p}^{2}=0.43$], showing that adaptation to the dot patterns resulted in a perceptual aftereffect. However, neither the main effect of dot quality [*F*(1, 22) = 1.52, *p* = 0.23, $\eta_{p}^{2}=0.06$], nor the interaction between dot quality and adaptor type [*F*(1, 22) = 1.11, *p* = 0.3, $\eta_{p}^{2}=0.05$] was significant. The three-way interaction between polarity, adaptor type and distortion level was also not significant [*F*(8, 176) = 0.37, *p* = 0.94, $\eta_{p}^{2}=0.02$]. These results suggest that the aftereffects are not affected strongly by the low-level properties of the constituent elements of the adaptor image. Finally, there was a significant main effect of distortion level [*F*(8, 176) = 103.61, *p* < 0.0001, $\eta_{p}^{2}=0.82$], and a significant interaction between distortion level and dot quality [*F*(8, 176) = 2.14, *p* = 0.03, $\eta_{p}^{2}=0.09$], but no interaction between distortion level and adaptor type [*F*(8, 176) = 0.88, *p* = 0.53, $\eta_{p}^{2}=0.04$].

**Figure 6:**
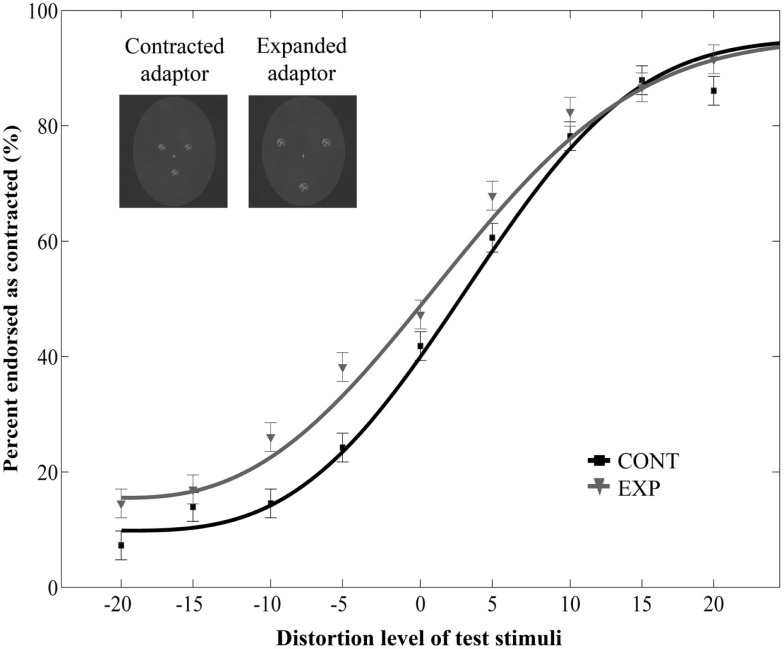
**Mean ratio of stimuli endorsed as contracted as a function of distortion level (% distorted) with low-contrast adaptor features consisting of visual noise**. Negative and positive distortion levels correspond to expanded and contracted target faces respectively. Results obtained by using the contracted (CONT) and expanded (EXP) dots consisting of visual noise.

However, the separate two-way repeated measures ANOVA on the data of Experiment 5 [with adaptor type (2) and distortion level (9) as within-subject factors] showed only a mild tendency of adaptor type effect [*F*(1, 10) = 2.36, *p* = 0.16, $\eta_{p}^{2}=0.19$; main effect of distortion level: *F*(8, 80) = 88.73, *p* < 0.0001, $\eta_{p}^{2}=0.9,$ no interaction between adaptor type and distortion level: *F*(8, 80) = 0.79, *p* = 0.61, $\eta_{p}^{2}=0.07,$ Figure 6]. The lack of significant main effect of adaptor type in the present experiment shows that lowering the contrast of the adaptor image reduces the amount of the aftereffect somewhat, even when the elements of the adaptor images are placed according to the basic face configuration. This result implies that the adaptation site is sensitive to changes affecting the low-level image properties, that is, the disruption of homogeneous brighter regions corresponding to eyes and mouth. The absence of any significant aftereffect in the separate ANOVA might be the consequence of the lower contrast between the brighter dots and the darker background, a possibility in line with the results of a previous study which showed that high-contrast faces generate stronger FDAEs than low-contrast ones (Yamashita et al., 2005).

## Discussion

In the present study, we demonstrated that FDAEs could be evoked by stimuli consisting of three dots arranged in a triangular position, corresponding to the position of usual facial features. This suggests that the processing sites underlying the FDAE are sensitive to the basic facial configuration and the fine spatial arrangement of the elements of the face (second-order relations) as well, even in the absence of realistic face parts.

One of the main questions is whether the aftereffect is due to the adaptation of low- or high-level visual areas, or both. One possibility is that the aftereffects originate from the early stages of visual processing, which are sensitive to the low-level visual properties of the image. A related assumption is that adaptation to the face-like patterns biased the response of low-level areas, and this bias propagated up the visual processing hierarchy, affecting the response of higher-level visual areas to the subsequently presented face-stimuli. Such “cross-level” (Xu et al., 2008) adaptation has been found previously with simple curved lines as adaptors, which not only affected the curvature judgments of target lines (low-level aftereffect) but the emotional expression decisions in real faces as well (high-level aftereffect; Xu et al., 2008). In case of low-level adaptation, however, due to the smaller receptive field sizes of the neurons we would expect the aftereffect to be sensitive to image size, whereas in Experiment 2 a significant aftereffect was observed in spite of the size difference between the adaptor and test stimuli. This result is in line with the previous finding that the FDAE tolerates large size differences between the adaptor and test images (Zhao and Chubb, 2001), and suggests the role of higher-level visual areas engaged in non-retinotopic visual processing and having a large degree of size-invariance.

However, these processing sites need not necessary be face-selective (Rhodes and Leopold, 2011). High-level, non-retinotopic aftereffects have been observed for general shape properties such as taper and aspect ratio (Suzuki and Cavanagh, 1998; Suzuki, 2005), which might have contributed to the aftereffects observed in the present study as well. On the other hand, in Experiment 3, we found that inverting the schematic face-like adaptor image eliminated the aftereffect entirely. Inverting a face is thought to interfere with face-specific configural processing mechanisms (see Introduction). Accordingly, a previous study showed that the inversion of the adaptor face (with the test face retaining its upright orientation) reduces the magnitude of the FDAE compared to any other combination of adaptor and test orientations (Watson and Clifford, 2003). Although the absence of aftereffect with an inverted adaptor in our study does not entirely exclude the possibility that a shape-generic mechanism can account for the aftereffect observed with upright adaptors, it strongly implies the involvement of face-specific mechanisms.

Human scalp electrophysiology and functional imaging studies provide considerable evidence that schematic and real faces share common or overlapping neural representations. The most widely studied electrophysiological correlate of face perception is the N170 event-related potential, which is larger for faces than for other object categories (Bentin et al., 1996; Rossion et al., 2000) and it is sensitive to manipulations that affect the canonical configuration of the face, such as inversion (e.g., Rossion et al., 2000) or scrambling of the face parts (e.g., George et al., 1996; Macchi Cassia et al., 2006). Note however, that results are mixed as to whether N170 is modulated (e.g., Scott and Nelson, 2006; Kaufmann and Schweinberger, 2012) or not (Mercure et al., 2008) by more subtle changes concerning the second-order relations of a face. The N170 evoked by schematic faces that lack realistic facial features but preserve the basic configuration is similar in amplitude to the N170 evoked by real face images (Sagiv and Bentin, 2001; Latinus and Taylor, 2006). Furthermore, schematic faces reduce the amplitude of the N170 to subsequently presented real faces, while schematic houses do not adapt the component (Eimer et al., 2011), suggesting that the same neural mechanisms underlie the perception of both types of faces.

Several functional imaging studies have shown that the FFA is sensitive to inversion (e.g., Yovel and Kanwisher, 2005; Mazard et al., 2006) and the disruption of first-order relations in upright faces, even in the absence of real face parts (Liu et al., 2009). Although an initial study did not show any differential sensitivity to features versus spacing between features (Yovel and Kanwisher, 2004), additional studies showed that the FFA (Rotshtein et al., 2007; Goffaux et al., 2009; Rhodes et al., 2009b) or a region adjacent to the FFA (Maurer et al., 2007) is sensitive to second-order relations. Schematic faces activate the FFA stronger than non-face objects, albeit less than real faces do (Tong et al., 2000). On the basis of these results, it is conceivable that the schematic face-like adaptors of the present study activated higher-level processing sites that are sensitive to the basic configuration of the facial features (first-order relations) and the spatial distance among the elements (second-order relations). Assuming that the schematic adaptors and the real test faces activated an overlapping set of neurons, adaptation might have desensitized the neurons responding to the schematic faces, which resulted in a shift of the overall population response in the opposite direction, biasing the representation of the test face. Conceptually, face aftereffects are usually interpreted in the framework of a multidimensional face-space (Valentine, 1991), in which individual variations in facial attributes are coded in relation to an average face or norm and adaptation shifts the norm toward the adaptor along the dimension that corresponds to the adapted attribute (e.g., Leopold et al., 2001; Robbins et al., 2007; Rhodes and Leopold, 2011). In this regard, it is plausible that prolonged exposure to the schematic adaptor resulted in a shift of the norm that is used to code second-order properties, which in turn then biased the representation of the test face away from the adaptor.

The fact that the observed FDAE was insensitive to the reversal of adaptor contrast supports this idea. Nevertheless, sensitivity to contrast reversal would not be entirely incompatible with the assertion that the aftereffect originates from higher-level visual areas either. This manipulation has been shown to affect the response of single neurons in the macaque inferior-temporal cortex (Perrett et al., 1984; Ito et al., 1994; Ohayon et al., 2012; but see Rolls and Baylis, 1986) as well as the BOLD response of the human fusiform gyrus to face images (George et al., 1999). Further, it has long been known that photographic negation, which reverses the contrast polarity of the image, is detrimental to face recognition (Galper, 1970; Galper and Hochberg, 1971). Under normal lighting conditions regions of the face corresponding to the eyes and mouth appear darker than their surroundings and the contrast reversal of the image reverses this pattern, making these areas lighter than the surrounding areas. Hence we would expect an aftereffect when the face-like adaptor image contains dark spots in the eye and mouth regions. Contrary to this, we observed an aftereffect with white dots on a gray (Experiment 1) or black (Experiment 4) background and also with black dots on a white background (Experiment 4). In a series of experiments, Kemp et al. (1990) showed that negation reduces sensitivity not only to the displacement of eyes in real faces, but to similar changes in stimuli consisting of three black dots arranged in a face-like configuration in a real facial surround. A more recent study using continuous flash supression (CFS), a form of binocular rivalry, showed that the mechanisms governing adults’ visual awareness are sensitive to inversion and negation of realistic face-stimuli as well as face-like patterns with three dark dots corresponding to the eyes and mouth, similar to our adaptor images (Stein et al., 2011). The authors conclude that even though CFS eliminates high-level face shape adaptation (Stein and Sterzer, 2011), a higher-level visual area such as the FFA could still play a role in these effects, based on the fact that activity in this area is informative of object category even if the stimulus itself is not consciously perceived (Sterzer et al., 2008). Whereas these studies point to the convergence of face-specific configural and contrast polarity cues, data from face adaptation studies show a somewhat different picture. The FDAE can be induced by both positive and negative polarity faces as well, and it is also selective to the polarity of the adaptor image (Yamashita et al., 2005). A more recent study has shown that the face identity aftereffect for famous faces is not affected by contrast reversal, as shown by the transfer of adaptation between positive and negative faces (Hills and Lewis, 2012). Our results, namely that schematic face-like images of opposite contrast polarity can be potent adaptors, is in line with these findings. This may be the result of dissociation between the coding of contrast polarity and configural properties at some levels of the visual system. The aftereffect in turn would depend on the adaptation of neurons tuned to the configural properties of the face, independently of contrast polarity. Another possibility, as suggested by Yamashita et al. (2005), is that positive and negative polarity faces adapt two separate mechanisms: face-specific and object-specific mechanisms respectively (see also Rhodes et al., 2004). However, in Experiment 3 we found that the adaptors consisting of white dots on a gray background, which approximate contrast negated faces, failed to induce an aftereffect when viewed upside-down. Since the effect of inversion is regarded as a hallmark of face-specific configural coding (Maurer et al., 2002; Rossion and Gauthier, 2002), this result seems to contradict the role of object-specific mechanism.

While it seems to be the case that the processing sites underlying these aftereffects are engaged in the coding of configural properties independently of contrast polarity, it does not necessarily follow that they are not sensitive to other lower-level image properties. In Experiment 5, we investigated adaptation to schematic face-like adaptors whose constituent elements had been replaced by visual noise. This manipulation disrupted the homogenous regions corresponding to the eyes and mouth. It also reduced the contrast between the blobs and their background, making them less salient compared to the white dots on a gray oval in Experiment 1. While the joint analysis of the two experiments did not show any difference, a separate analysis of data solely from Experiment 5 showed only a minor tendency for FDAE. This might be the result of the reduced contrast between the internal elements and their background. Higher-level areas of the human visual cortex show less sensitivity to contrast changes then lower level ones, and this is trend is more pronounced for faces than objects (Avidan et al., 2002). On the other hand, contrast strength has been shown to affect the FDAE, as high-contrast faces evoke stronger aftereffects than low-contrast ones (Yamashita et al., 2005). Therefore, our results may reflect certain contrast sensitivity of the adaptation sites underlying the FDAE, although the origin of this effect remains to be explored.

Finally, there are some important issues worth considering. First, we observed that participants tended to perceive test faces to be more expanded than contracted. As can be seen in Figure 2 of Experiment 1, at 0% distortion, the percentage of contracted ratings remained below 50% even in the expanded adaptor condition. While the source of this effect is not clear, one factor that might have contributed to this effect is the asymmetrical sensitivity to different directions of distortion. Previous studies investigating the sensitivity to changes affecting facial configuration have shown that people are more sensitive to inwards than outwards shifts of the eyes (Haig, 1984; Kemp et al., 1990). This might have counteracted the aftereffect to the extent that the veridical face was more often reported as “expanded” than “contracted.”

A further question to be addressed is whether the aftereffects in the present study are comparable in strength to the aftereffects obtained by real face adaptors. Although the present study only employed schematic face-like adaptors, the stimulus material partially (the Angelina Jolie face-line was used in both studies) overlapped with a previous study of our lab (Zimmer and Kovács, 2011a). The comparison of results (see Figure 2 of Zimmer and Kovács, 2011a) shows that the aftereffects evoked by schematic adaptors are smaller in magnitude than the ones observed with real face adaptors (note however, that the methodological differences, such as the slightly shorter adaptation duration of the present study, limit the validity of this comparison). This difference suggests that besides configural processing, the adaptation of neural pools engaged in feature encoding also contributes to the FDAEs observed with real face adaptors.

In summary, we found that FDAE s can be evoked by adaptation to stimuli that only retain the basic configuration of a real face: three dots in the location of the eyes and the mouth, embedded in an oval. Aftereffects were also observed when the adaptor and test faces differed in size, suggesting that the perceptual bias depends at least in part on the adaptation of higher-level neural populations. However, with adaptors turned upside-down, we did not observe any aftereffects, which might be due to the disruption of face-specific configural coding. The aftereffects did not depend on the contrast polarity of the adaptor image either. On the other hand, replacing these elements with blobs consisting of visual noise reduced the aftereffects, which might be the consequence of the low-contrast of the elements. Thus, while the adaptation sites seem to be engaged in the coding of facial configuration independently of contrast polarity, they also appear to be sensitive to contrast manipulations affecting the saliency of the inner elements to a certain degree.

## Conflict of Interest Statement

The authors declare that the research was conducted in the absence of any commercial or financial relationships that could be construed as a potential conflict of interest.
